# The trend of direct medical costs and associated factors in patients with chronic hepatitis B in Guangzhou, China: an eight-year retrospective cohort study

**DOI:** 10.1186/s12911-021-01429-6

**Published:** 2021-07-30

**Authors:** Shuo Yang, Ge Chen, Yueping Li, Guanhai Li, Yingfang Liang, Feng Zhou, Shudong Zhou, Yi Yang, Weidong Jia, Yanhui Gao, Yue Chen

**Affiliations:** 1 Department of Epidemiology and Medical Statistics, School of Public Health, Guangdong Pharmaceutical University, Guangzhou, Guangdong China; 2 Guangzhou Eighth People’s Hospital, Guangzhou, Guangdong China; 3 Department of Public Health and Preventive Medicine, School of Medicine, Jinan University, Guangzhou, Guangdong China; 4 School of Epidemiology and Public Health, Faculty of Medicine, University of Ottawa, Ottawa, ON Canada

**Keywords:** Chronic hepatitis B, Direct medical costs, Generalized estimating equations, Quantile regression

## Abstract

**Background:**

Although the expenses of liver cirrhosis are covered by a critical illness fund under the current health insurance program in China, the medical costs associated with hepatitis B virus (HBV) related diseases is not well addressed. In order to provide evidence to address the problem, we investigated the trend of direct medical costs and associated factors in patients with chronic HBV infection.

**Methods:**

A retrospective cohort study of 65,175 outpatients and 12,649 inpatients was conducted using a hospital information system database for the period from 2008 to 2015. Generalized estimating equations (GEE) were applied to explore associations between annual direct medical costs and corresponding factors, meanwhile quantile regression models were used to evaluate the effect of treatment modes on different quantiles of annual direct medical costs stratified by medical insurances.

**Results:**

The direct medical costs increased with time, but the proportion of antiviral costs decreased with CHB progression. Antiviral costs accounted 54.61% of total direct medical costs for outpatients, but only 6.17% for inpatients. Non-antiviral medicine costs (46.06%) and lab tests costs (23.63%) accounted for the majority of the cost for inpatients. The direct medical costs were positively associated with CHB progression and hospitalization days in inpatients. The direct medical costs were the highest in outpatients with medical insurance and in inpatients with free medical service, and treatment modes had different effects on the direct medical costs in patients with and without medical insurance.

**Conclusions:**

CHB patients had a heavy economic burden in Guangzhou, China, which increased over time, which were influenced by payment mode and treatment mode.

## Background

Hepatitis B virus (HBV) infection is a major global health concern [1]. In China, the overall annual incidence of hepatitis B was 80.63 per 10 million during the period from 2004 to 2013 [2], and a recent systematic review of global HBV infection indicated that China was an intermediate endemic area for the infection, with a combined prevalence of HBV infection of 5.7% [3]. China has the largest number of HBV infected people in the world, and there are approximately 76 million HBV carriers among the populations under the age of 60 years [4].

Patients infected with HBV are at significant risk for developing chronic hepatitis B (CHB), compensated cirrhosis, decompensated cirrhosis, and primary hepatocellular carcinoma (HCC) [5]. CHB and its related diseases pose a substantial economic burden on patients and their families [6, 7], and medical costs comprise the majority portion of the total expenditures and increase with disease progression [6, 8–13].

However, there was high heterogeneity for the estimates of medical costs due to HBV infection from previous studies [8–16] and cross-sectional or short term data did not provide a trajectory of the costs over time. This study aimed to estimate the direct medical costs due to HBV infection and its complications, costs components and associated factors based on data from a real-world retrospective cohort of CHB patients for the period from January 1, 2008 to December 31, 2015 in Guangzhou, China. The results can be utilized in cost-effectiveness evaluation of treatment and help healthcare providers and policy makers for their efforts in improving patients' health conditions and reducing medical costs.

## Methods

### Study design and study population

Two retrospective cohorts of outpatients and inpatients were created using the electronic medical records from the hospital information system (HIS) of the Guangzhou Eighth People’s Hospital, the largest specialized infectious disease hospital in Guangdong Province, China. This cohort included inpatients and outpatients with HBV related conditions including CHB, cirrhosis (including compensated and decompensated cirrhosis) and HCC during the period from January 1, 2008 to December 31, 2015, who were identified according to the International Classification of Disease, 10^th^ Revision (ICD-10) codes (CHB: B18.0 and B18.1, cirrhosis: K74.601 and K74.602, HCC: K76.814). Patients co-infected with hepatitis A, hepatitis C, hepatitis D, hepatitis E, HIV or cytomegalovirus (CMV)were excluded from this study. Patients admitted to the hospital due to pregnancy or other diseases including glomerulonephritis, uremia, metabolic syndrome, tumor, sever cardiovascular diseases were also excluded.

### Study variables

Information on demographics, health insurance status, clinical diagnosis, and medications prescribed and their costs were extracted from patients’ medical records. Direct medical costs in this study were classified into the following categories: (1) laboratory tests and imaging examinations, (2) antiviral therapy, (3) other treatment of HBV infection such as hepatoprotective drugs, traditional Chinese medication, (4) bed and nursing costs (for inpatients only) and (5) other costs including registration and consultation costs (for outpatients only), radiation therapy, anesthesia, surgery and blood transfusion (for inpatients only). The total annual direct medical costs for each patient were the sum of all these costs every year. Types of treatment were anti-viral drug (yes/no), hepatoprotective drug (yes/no), traditional Chinese medication (yes/no) and anti-fibrotic medication (yes/no). “Yes” means that a patient received a prescription of specific drugs in the year of visit/admission. Anti-viral drugs included Interferon (IFN), lamivudine (LAM), adefovir dipivoxil (ADV), entecavir (ETV), telbivudine (LdT) and tenofovir (TDF). Patients were grouped into the following categories according to health insurance status: free medical service, medical insurance, self-payment and others.

### Statistical analysis

The direct medical costs per person per year were taken as the analysis unit. Mean, median and upper and lower quartiles of annual direct medical costs were calculated, and the costs were expressed in RMB in the year of 2015 and were adjusted by a discount rate of 5% per year. Average annual direct medical costs, costs components and corresponding proportions of costs components in total direct medical costs were also calculated for inpatients and outpatients by different disease stages.

Because of extremely positive partial peak distribution and individual correlation of medical costs between multiple visits/admissions for individual patients, generalized estimating equations (GEE) [17] with log link function and gamma error distribution were applied to explore the associations between determinants and direct medical costs. Quantile regression models [18] were used to assess the effect of treatment modalities on direct medical costs for patients according to medical insurance status. Quantile regression makes no requirement for the distribution of random errors, and is particularly effective when the distribution is asymmetric, thick-tailed, or deleted. The statistical analyses were conducted separately for outpatients and inpatients by using SAS 9.4 (SAS Institute, Cray, NC).

## Results

### General characteristics of the study population

**Fig. 1:**
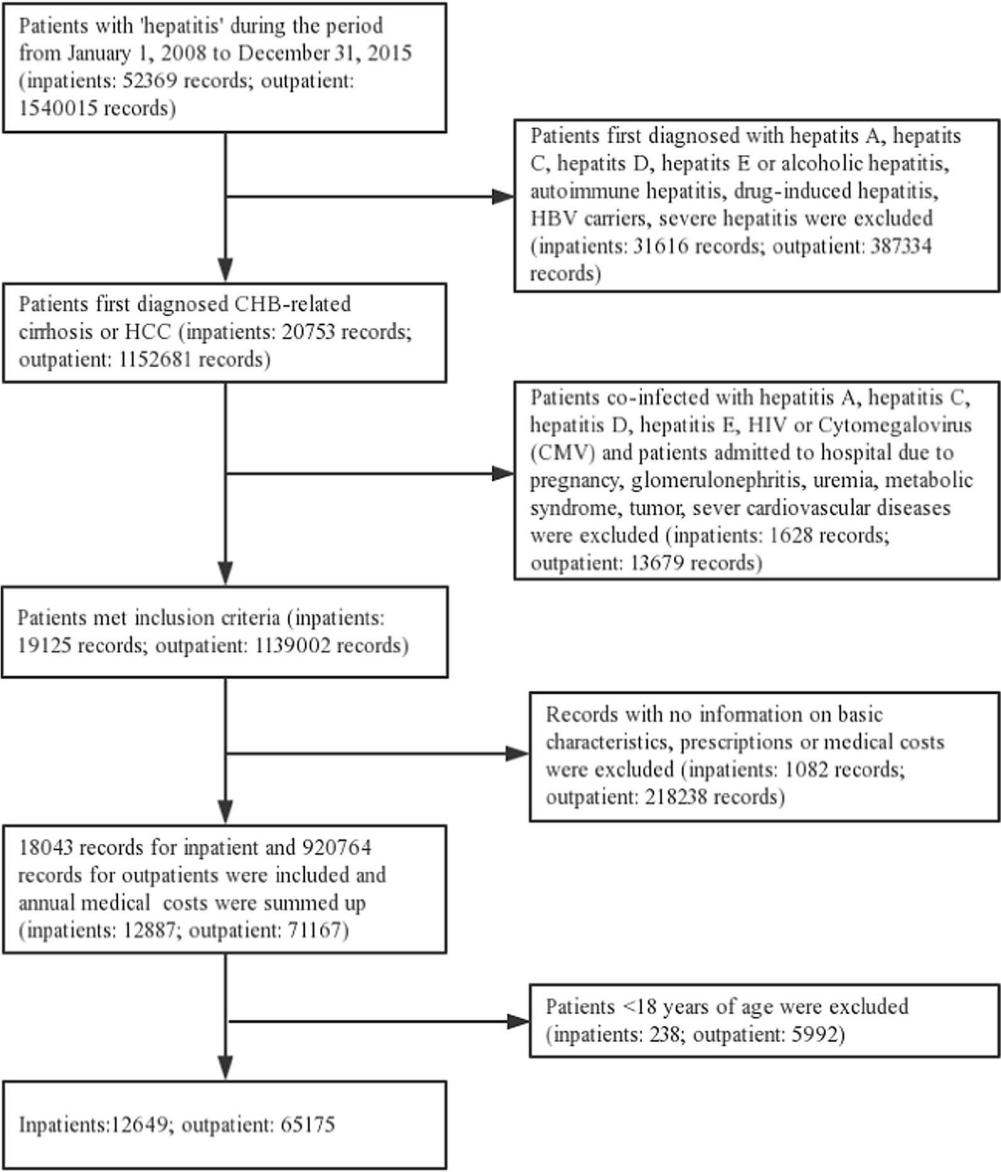
Flow chart of records selection

A total of 65,175 outpatients and 12,649 inpatients were retrieved from the Guangzhou Eighth People’s Hospital database for the study period between January 1, 2008 and December 31, 2015 (Fig. 1). General characteristics of the outpatients and the inpatients by disease stages were detailed in Table 1. Approximate 90% outpatient visits and 57% inpatients admissions were due to CHB, 9% and 27% were due to cirrhosis, and 1% and 16% were due to HCC. About 75% of these patients were male and the proportion of patients aged 40 to 60 accounted for approximately 50%. Self-payment and medical insurance were the main payment modes. Table 1 also shows the proportions of patients received prescriptions for antiviral drugs, hepatoprotective drugs, traditional Chinese medicine and anti-fibrotic medicine for outpatients and inpatients.

**Table 1:** Characteristics and medication use of outpatients and inpatients [N (%)]

Characteristics	Outpatients				Inpatients			
	CHB	Cirrhosis	HCC	Total	CHB	Cirrhosis	HCC	Total
N	58,857 (90.31)	5610 (8.61)	708 (1.09)	65,175 (100.00)	7224 (57.11)	3449 (27.27)	1976 (15.62)	12,649 (100.00)
Age (years)^a^	35.07 ± 12.24	48.90 ± 12.95	52.62 ± 13.08	36.45 ± 13.02	34.11 ± 11.54	49.72 ± 12.66	52.02 ± 12.53	41.17 ± 14.53
Gender^b^								
Male	41,816 (71.08)	4241 (75.61)	603 (85.17)	46,660 (71.62)	5287 (73.19)	2651 (76.86)	1719 (86.99)	9657 (76.35)
Female	17,015 (28.92)	1368 (24.39)	105 (14.83)	18,488 (28.38)	1937 (26.81)	798 (23.14)	257 (13.01)	2992 (23.65)
Payment mode								
Self-payment	39,481 (67.08)	3958 (70.55)	607 (85.73)	44,046 (67.58)	4756 (65.84)	2379 (68.98)	1393 (70.50)	8528 (67.42)
Medical insurance	15,419 (26.20)	1314 (23.42)	75 (10.59)	16,808 (25.79)	2128 (29.46)	910 (26.38)	493 (24.95)	3531 (27.92)
Free medical service	3951 (6.71)	338 (6.02)	26 (3.67)	4315 (6.62)	301 (4.17)	130 (3.77)	74 (3.74)	505 (3.99)
Others^c^	6 (0.01)	0 (0.00)	0 (0.00)	6 (0.01)	39 (0.54)	30 (0.87)	16 (0.81)	85 (0.67)
Antiviral medicine								
Yes^d^	25,525 (43.37)	2803 (49.96)	172 (24.29)	28,500 (43.73)	4036 (55.87)	2221 (64.40)	1031 (52.18)	7288 (57.62)
No	33,332 (56.63)	2807 (50.04)	536 (75.71)	36,675 (56.27)	3188 (44.13)	1228 (35.60)	945 (47.82)	5361 (42.38)
IFN								
Yes^d^	4092 (6.95)	44 (0.78)	0 (0.00)	4136 (6.35)	1768 (24.47)	79 (2.29)	1 (0.05)	1848 (14.61)
No	54,765 (93.05)	5566 (99.22)	708 (100.00)	61,039 (93.65)	5456 (75.53)	3370 (97.71)	1975 (99.95)	10,801 (85.39)
NAs								
Yes^d^	22,811 (38.76)	2778 (49.52)	172 (24.29)	25,761 (39.53)	2402 (33.25)	2148 (62.28)	1030 (52.13)	5580 (44.11)
No	36,046 (61.24)	2832 (50.48)	536 (75.71)	39,414 (60.47)	4822 (66.75)	1301 (37.72)	946 (47.87)	7069 (55.89)
Hepatoprotective drugs								
Yes^d^	32,247 (54.79)	2447 (43.62)	207 (29.24)	34,901 (53.55)	6686 (92.55)	3338 (96.78)	1917 (97.01)	11,941 (94.40)
No	26,610 (45.21)	3163 (56.38)	501 (70.76)	30,274 (46.45)	538 (7.45)	111 (3.22)	59 (2.99)	708 (5.60)
Chinese medicine								
Yes^d^	26,172 (44.47)	3068 (54.69)	255 (36.02)	29,495 (45.26)	3741 (51.79)	2302 (66.74)	1264 (63.97)	7307 (57.77)
No	32,685 (55.53)	2542 (45.31)	453 (63.98)	35,680 (54.74)	3483 (48.21)	1147 (33.26)	712 (36.03)	5342 (42.23)
Anti-fibrotic medicine								
Yes^d^	9142 (15.53)	3298 (58.79)	214 (30.23)	12,654 (19.42)	936 (12.96)	1895 (54.94)	858 (43.42)	3689 (29.16)
No	49,715 (84.47)	2312 (41.21)	494 (69.77)	52,521 (80.58)	6288 (87.04)	1554 (45.06)	1118 (56.58)	8960 (70.84)
Visiting times^a^	4 (2–14)	4 (2–15)	1 (1–3)	4 (2–14)	1 (1–1)	1 (1–2)	1 (1–1)	1 (1–1)

^a^Mean ± STD

^b^Gender: outpatients have 27 gender missing values

^c^Others: commercial insurance and cooperative medical service, etc.

^d^Yes: patients received prescription for corresponding drugs in this year

### Average annual direct medical costs and costs components from 2008 to 2015

The average annual direct medical costs for outpatients and inpatients were 3731.05 and 12,967.77 RMB for CHB; 5871.55 and 23,869.57 RMB for cirrhosis; and 3734.82 and 27,996.54 RMB for HCC, respectively. Table 2 showed the components of average annual direct medical costs for patients with different disease stages. For outpatients, use of antiviral drugs (54.61%) was the costliest component, followed by non-antiviral medications (28.76%, including 9.80% of hepatoprotective drugs, 11.00% of traditional Chinese medicine and 8.71% anti-fibrotic drugs). For inpatients, use of non-antiviral medications contributed 46.06% (including 15.68% of hepatoprotective drugs, 2.92% of traditional Chinese medications and 1.49% anti-fibrotic drugs) and lab tests and imaging examinations contributed 23.63% of the total costs. Antiviral medication costs accounted for only 6.17% of the total costs for inpatients.

**Table 2:** Average annual direct medical costs and cost components by disease states (RMB)

Disease stages	Total costs	Lab tests and imaging examinations costs (%)	Antiviral therapy costs (%)	Other medicine costs (%)	Bed and nursing costs (%)	Other costs (%)	Hepatoprotective drugs costs (%)	Chinese medicine costs (%)	Anti-fibrotic medicine costs (%)
Outpatients									
CHB	3731.05	612.59 (16.42)	2115.80 (56.71)	973.74 (26.10)	–	26.77 (0.72)	402.26 (10.78)	321.68 (8.62)	192.66 (5.16)
Cirrhosis	5871.55	791.89 (13.49)	2762.03 (47.04)	2280.05 (38.83)	–	35.57 (0.61)	352.18 (6.00)	1171.40 (19.95)	1317.66 (22.44)
HCC	3734.82	736.14 (19.71)	1347.16 (36.07)	1597.73 (42.78)	–	51.37 (1.38)	230.27 (6.17)	897.42 (24.03)	782.29 (20.95)
All	4019.52	638.01 (15.87)	2195.07 (54.61)	1156.11 (28.76)	–	28.20 (0.70)	393.77 (9.80)	442.03 (11.00)	350.25 (8.71)
Inpatients									
CHB	12,967.77	3582.27 (27.62)	1248.97 (9.63)	5549.54 (42.79)	1185.32 (9.14)	1401.66 (10.81)	2827.57 (21.80)	274.13 (2.11)	80.71 (0.62)
Cirrhosis	23,869.57	5352.03 (22.42)	1223.75 (5.13)	11,798.04 (49.43)	1479.03 (6.20)	4016.72 (16.83)	3352.95 (14.05)	781.21 (3.27)	534.99 (2.24)
HCC	27,996.54	5513.45 (19.69)	765.67 (2.73)	12,775.57 (45.63)	1294.78 (4.62)	7647.07 (27.31)	2594.88 (9.27)	993.19 (3.55)	441.90 (1.58)
All	18,801.96	4443.79 (23.63)	1159.99 (6.17)	8659.90 (46.06)	1292.78 (6.87)	3245.50 (17.26)	2947.68 (15.68)	548.87 (2.92)	279.21 (1.49)

**Fig. 2:**
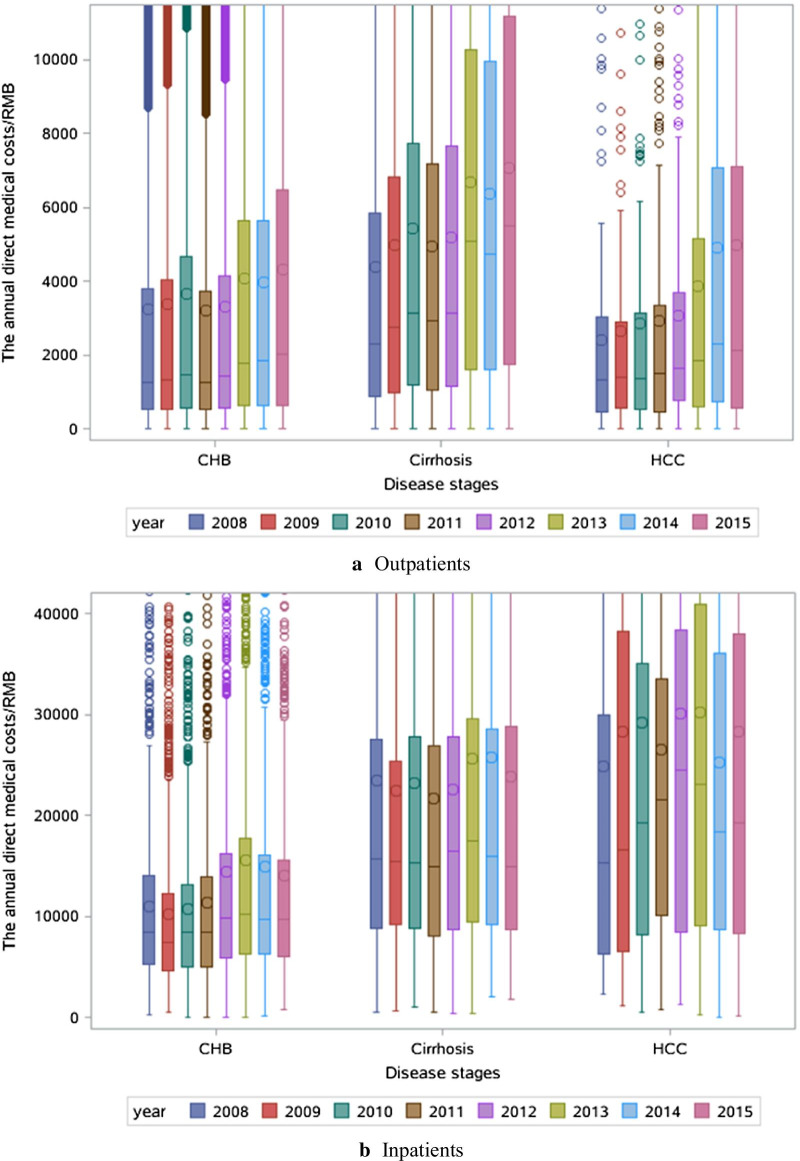
Annual direct medical costs by disease stage in outpatients and inpatients in 2008–2015

**Fig. 3:**
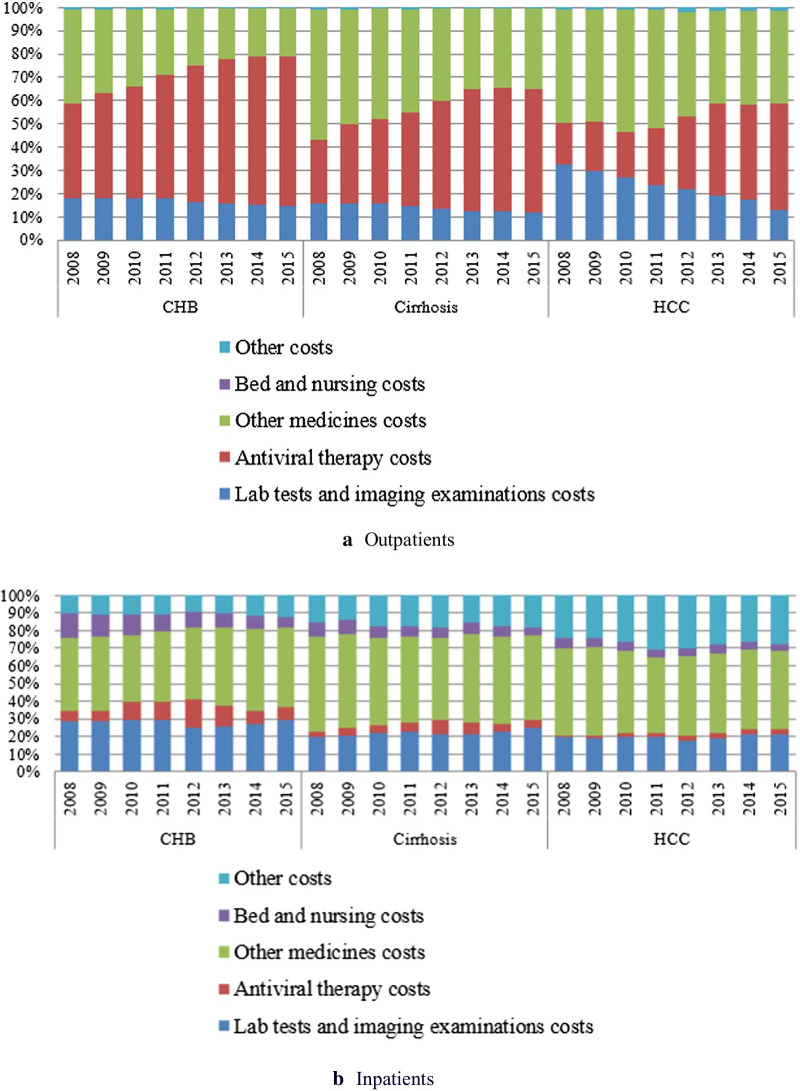
Components of medical costs by disease stage in outpatients and inpatients in 2008–2015

The direct medical costs were mostly increasing from the year of 2008 to 2015 for all the patients (Fig. 2). The proportion of antiviral drug use was decreasing with the disease stage (Fig. 3). The costliest component was antiviral drug use for outpatients and use of non-antiviral drugs for inpatients. Among outpatients, the proportion of antiviral drug use increased, whereas the proportion of non-antiviral drug use and lab test decreased over time.

### Associated factors for annual direct medical costs

Table 3 showed the factors associated with annual direct medical costs in outpatients and inpatients. Male and older patients had significantly higher costs as compared with female and younger patients (*P* < 0.0001). For outpatients, those with medical insurance had the highest costs, followed by those who paid out-of-pocket and those with free medical service. For inpatients, those with free medical service had the highest costs, followed by patients with medical insurance and those who paid out-of-pocket. Antiviral therapy [β(SE) = 1.68(0.01), *P* < 0.0001], hepatoprotective drugs [β(SE) = 1.68(0.01), *P* < 0.0001], traditional Chinese medication [β(SE) = 0.22(0.01), *P* < 0.0001] and anti-fibrotic medication [β(SE) = 0.55(0.01), *P* < 0.0001] significantly increased the direct medical costs for outpatients. Similarly, antiviral therapy [β(SE) = 0.17(0.01), *P* < 0.0001], hepatoprotective drugs [β(SE) = 0.25(0.01), *P* < 0.0001], traditional Chinese medication [β(SE) = 0.17(0.01), *P* < 0.0001] significantly increased the direct medical costs for inpatients. The direct medical costs were positively associated with the progression of HBV infection and hospitalization days in inpatients (*P* < 0.0001).

**Table 3:** Characteristics and factors associated with annual direct medical costs among outpatients and inpatients using GEE with log link and gamma distribution

Factors	Outpatients					Inpatients				
	Cost (mean)	Cost (M(Q_1_–Q_3_))	*β*	SE^d^	*P*	Cost (mean)	Cost (M(Q_1_–Q_3_))	*β*	SE^d^	*P*
Gender^a^										
Male	4198.92	1909.98 (664.50–5813.24)	Ref			19,449.20	12,004.35 (6765.12–22,922.24)	Ref		
Female	3530.35	1463.41 (553.91–4616.10)	–0.07	0.01	< 0.0001	16,701.86	10,604.72 (6300.63–19,184.97)	–0.06	0.01	< 0.0001
Age										
18–29	3638.45	1398.60 (541.19–4592.04)	Ref			12,173.28	8263.53 (5078.25–13,815.65)	Ref		
30–39	4171.64	1802.43 (636.68–5708.63)	0.03	0.01	0.0068	15,866.61	10,525.78 (6150.71–18,696.58)	0.11	0.02	< 0.0001
40–49	4336.78	2210.05 (734.33–6163.25)	–0.01	0.01	0.8982	21,198.46	13,456.04 (7303.28–25,995.43)	0.22	0.02	< 0.0001
50–59	4185.27	2093.56 (737.78–5985.38)	–0.05	0.01	0.0008	23,465.83	15,388.61 (8448.31–29,266.38)	0.24	0.02	< 0.0001
≥ 60	3847.18	1893.67 (667.02–5364.38)	–0.10	0.02	< 0.0001	26,068.35	16,874.79 (9145.88 – 31,415.35)	0.28	0.02	< 0.0001
Payment mode										
Self-payment	3712.91	1559.49 (588.49–4887.64)	ref			17,784.66	11,168.72 (6287.71–21,266.31)	Ref		
Medical insurance	5066.04	2862.03 (882.33–7450.20)	0.11	0.01	< 0.0001	20,808.66	12,406.73 (7273.45–23,788.23)	–0.04	0.01	0.0018
Free medical service	2784.96	1140.70 (445.45–3205.32)	–0.19	0.02	< 0.0001	21,428.33	14,006.00 (8526.01–25,144.22)	–0.14	0.03	< 0.0001
Others^b^	852.51	706.95 (403.26–1084.77)	–0.14	0.13	0.2839	13,166.87	8786.42 (5699.22–17,312.19)	–0.09	0.07	0. 1810
Antiviral medicine										
No	1475.46	771.42 (402.67–1692.61)	ref			14,125.19	8132.11 (4841.38–15,879.36)	Ref		
Yes^c^	7020.08	5357.05 (2301.01–9959.00)	1.68	0.01	< 0.0001	22,221.89	14,401.12 (8643.15–26,762.65)	0.17	0.01	< 0.0001
Hepatoprotective drugs										
No	3783.10	778.77 (408.76–1706.41)	ref			6774.95	4308.40 (3100.17–7352.18)	Ref		
Yes^c^	4260.31	5356.04 (2306.41–9944.59)	0.51	0.01	< 0.0001	19,695.67	12,342.43 (7257.06–23,087.47)	0.25	0.02	< 0.0001
No	3386.25	1313.54 (517.40–4527.89)	Ref			13,039.13	8274.40 (4949.98–15,263.26)	ref		
Yes^c^	5239.27	2859.98 (1098.96–7249.16)	0.22	0.01	< 0.0001	23,146.26	14,760.32 (8639.26–28,007.82)	0.17	0.01	< 0.0001
Anti-fibrotic medicine										
No	3489.43	1422.17 (555.26–4543.92)	Ref			16,574.29	10,036.57 (5880.35–18,612.07)	Ref		
Yes^c^	6763.95	4843.03 (1937.49–9969.05)	0.55	0.01	< 0.0001	24,049.95	16,327.63 (9261.82–29,651.38)	–0.02	0.01	0.1162
Disease stages										
CHB	3731.05	1570.04 (587.63–4954.84)	Ref			12,967.77	9094.66 (5550.94–15,113.85)	Ref		
Cirrhosis	5871.55	3823.50 (1285.07–9027.62)	0.14	0.01	< 0.0001	23,869.57	15,703.35 (8847.61–28,033.55)	0.40	0.03	< 0.0001
HCC	3734.82	1676.06 (610.75–4737.08)	0.10	0.05	0.0443	27,996.54	20,271.02 (8274.40–36,742.64)	0.62	0.03	< 0.0001
Length of stay (days)										
< 10	–	–	–	–	–	6633.50	5304.68 (3879.56–7584.06)	ref		
10–20	–	–	–	–	–	13,584.70	10,574.83 (7767.96–15,979.52)	0.61	0.01	< 0.0001
> 20	–	–	–	–	–	33,436.70	23,685.30 (15,248.07–41,328.95)	1.47	0.02	< 0.0001

^a^Gender: 60 were missing for outpatients

^b^Others: commercial insurance and cooperative medical service, etc.

^c^Yes: patients received prescription for corresponding drugs

^d^SE: standard error

**Fig. 4:**
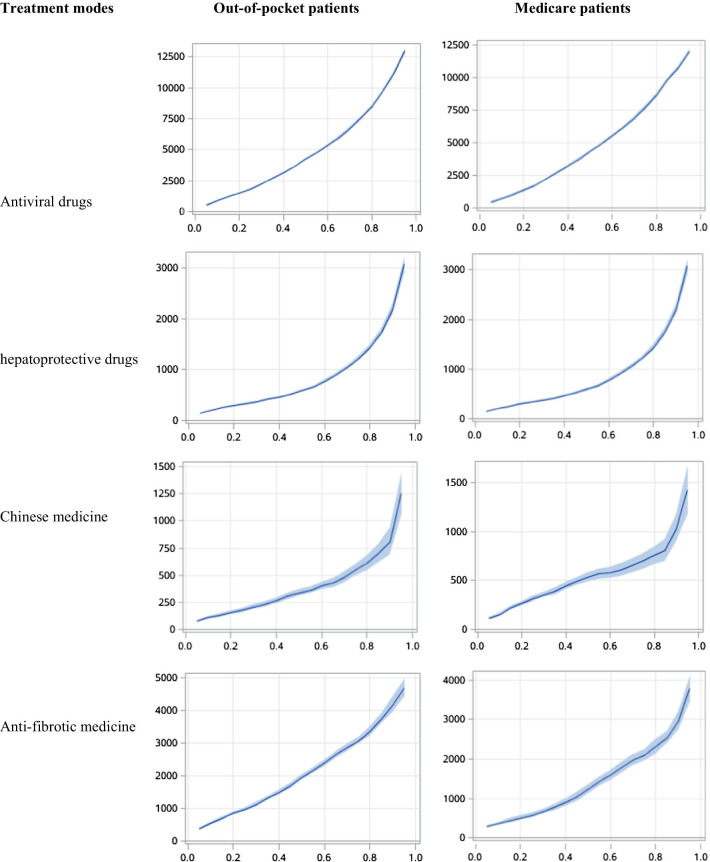
Impact of different treatment modes on direct medical costs in outpatients: quantile regression

**Fig. 5:**
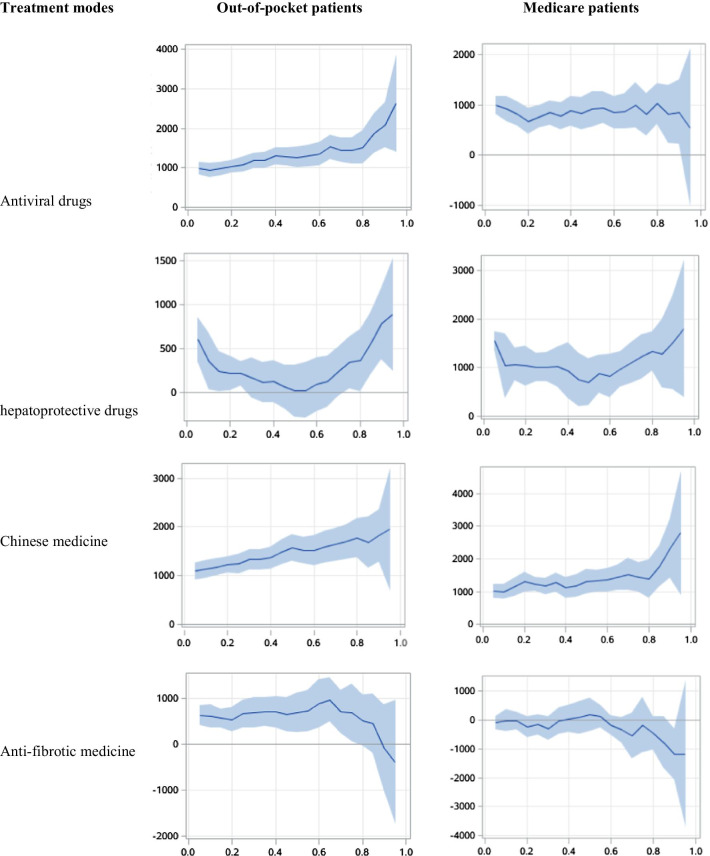
Impact of different treatment modes on direct medical costs in inpatients: quantile regression

Figures 4 and 5 showed the results from quantile regression models for treatment modes associated with direct medical costs. In outpatients, the utilization of antiviral drugs, hepatoprotective drugs, traditional Chinese medications and anti-fibrosis drugs increased the direct medical costs of patients with or without medical insurance, and it was more so for patients with a high level of medical costs. In inpatients, the utilization of antiviral drugs significantly increased the direct medical costs. The 25%, 50% and 75% of quantile regression coefficients for antiviral treatment associated with the direct medical costs were 1081.45, 1264.65 and 1435.69 for out-of-pocket inpatients, respectively, as compared with 771.33, 920.32 and 809.75 insured inpatients. However, the influences of hepatoprotective drugs, traditional Chinese medication and anti-fibrosis drugs on the direct medical costs were similar for inpatients with and without medical insurance.

## Discussion

Our study found that the average annual direct medical costs for patients with HBV related diseases in Guangzhou were substantial and the annual direct medical costs were increasing for both outpatients and inpatients from 2008 to 2015. Guangzhou is the capital city of Guangdong Province, Southeast China, is the forefront of China’s economic and social development. According to the annual report of the bureau of statistics in Guangzhou, the disposable personal income was 46,734.60 RMB in 2015 [19]. As for CHB patients, the direct medical costs of the three disease stages accounted for 7.98–12.56% of individual income annually on average for outpatients and 27.75–59.91% for inpatients in Guangzhou, which is slightly higher than results of 5–40% from a study from Beijing in 2012 [20].

Our results indicated that treatment mode was an important determinant of annual direct medical costs for CHB patients. Antiviral therapy is the most key to favorable prognosis for CHB-related disease patients, especially in the early stages of HBV infection [21]. The annual direct medical costs for patients with antiviral therapy were consistently higher than those without antiviral treatment (fivefold for outpatients vs 1.2-fold for inpatients), and the annual costs of antiviral medications and its percentage of the total medical costs descended with disease progression. The antiviral medications accounted for 65.50% of the medical costs for CHB outpatients but only 45.75% for HCC outpatients. Patients with cirrhosis and HCC utilized more medical resources other than antiviral medications. Compared with outpatients, the proportion of antiviral treatment costs in inpatients were lower and only accounted for a small percentage of the direct medical costs for each disease stages, while other medications and lab tests and imaging examinations made up a major percentage of the costs.

CHB patients acquire a higher proportion of antiviral treatment in the early disease stage to prevent the progression of liver diseases. However, this study shows that the antiviral utilization rate was not high, only 43.73% for outpatients and 57.62% for inpatients. A study from five European countries (including Germany, France, Turkey, Poland and Romania) showed that the antiviral utilization rate was 44.75% [22]. Similarly, a study from Beijing reported the antiviral utilization rate was 50.00% for outpatients and 61.80% for inpatients [20]. The reasons for the low antiviral utilization rate should be further studied.

To effectively protect the liver of patients is one challenge for the medical and pharmaceutical professions [23, 24]. Available chemical drugs used for the treatment of liver disease could have a variety of adverse effects and damage the liver. Hepatoprotective drugs have the function of protecting liver, reducing transaminase and enhancing immunocompetence, thus it has been recommended as an adjuvant therapy for the treatment of chronic hepatitis B [4, 25]. In our study, 53.55% of outpatients and 94.40% received hepatoprotective drugs treatment, and 19.42% of outpatients and 29.16% received anti-fibrosis drugs. Hepatoprotective drugs of traditional Chinese medicine (TCM) [4] is popular in China, especially for CHB patients who fail to recover and seek alternative therapy [8, 26]. Our study showed that 45.26% of outpatients and 57.77% of inpatients received TCM treatment.

Health insurance status is an important influencing factor for medical costs [20, 27, 28]. The direct medical costs were the highest for outpatients with medical insurance and for inpatients with free medical service. Quantile regression showed that the effect of treatment mode on medical expenses was different for patients with different medical insurance status, especially for inpatients since free medical service and health insurance policies often provide a higher proportion of reimbursement for inpatients than for outpatients. Patients are motivated to be hospitalized to receive antiviral therapy or to have expensive tests for acquiring more reimbursement. Insurance policies should be modified to avoid the waste of medical resources.

In this study, patients with medical insurance and those out-of-pocket were accounted for 67.76% and 26.13% of the total population, respectively. There are still medical insurance policy differences for the different CHB patients now. Since 2008, health insurance policy has experienced a series of changes in Guangzhou. The Outpatient Medical Insurance Fund Policy was implemented in Guangzhou on August 1, 2008, that provided monthly quota payment for patients. Until October 1, 2013, both urban and rural residents were paid with CHB patients 100 RMB per month by this Fund. The payment increased to 400 RMB per month on January 1, 2015. Our previous studies have shown that those policy changes could promote the utilization of antiviral drugs in patients with CHB [29].

In the long run, the insurance system should be improved to narrow the gap between different medical insurance policy, and it is important to gradually enlarge the outpatient coverage and improve utilization of antiviral drugs. Meanwhile, HBV diagnosis, prevention and treatment behavior should be regulated and strengthened to reduce the economic burden of CHB patients, safeguarding their health interests.

This was a retrospective cohort study based on medical records of CHB patients from one hospital and has some limitations. First, patients might visited other hospitals or pharmacies and the medical costs might be underestimated. Second, it is difficult to distinguish treatment-naive patients with treatment-experienced patients, and therefore, switch or add-on treatments were not considered. Third, the retrospective study design could result in incomplete information. Fourth, our data only came from one hospital. However, Guangzhou Eighth People’s Hospital is the earliest and the only specialized liver disease hospital in Guangdong, and 8-year retrospective cohort of CHB showed stable and adequate cases in this hospital. Except hepatitis B-related HCC patients, it is to a certain extent representative for patients with CHB and cirrhosis. Other potential confounding factors such as education, income and severity of the disease were not taken into account in this study.

## Conclusion

CHB patients had a heavy economic burden that continued to be intensified. Annual direct medical costs for CHB patients increased significantly as the disease stage progressed although the proportion of the antiviral medication costs decreased. Antiviral therapy costed the most for CHB outpatients but for inpatients, non-antiviral medications and lab tests and imaging examinations were main components of the direct medical costs. Treatment patterns and medical insurance policies were main influencing factors of the annual direct medical costs. Early antiviral treatment could reduce the economic burden of CHB patients, and rational drug utilization and expanding medical insurance coverage might reduce the direct medical costs for CHB patients.

## Data Availability

The datasets used and/or analyzed during the current study are available from the corresponding author on reasonable request.

## Abbreviations

HBV: Hepatitis B virus
CHB: Chronic hepatitis B
HCC: Hepatocellular carcinoma
GEE: Generalized estimating equations
HIS: Hospital information system
ICD: International classification of disease
CMV: Cytomegalovirus
IFN: Interferon
LAM: Lamivudine
ADV: Adefovir dipivoxil
ETV: Entecavir
LdT: Telbivudine
TDF: Tenofovir
TCM: Traditional Chinese medicine
DRGs: Diagnosis related groups
